# Expression and Potential Roles of *HLA-G* in Human Spermatogenesis and Early Embryonic Development

**DOI:** 10.1371/journal.pone.0092889

**Published:** 2014-03-25

**Authors:** Gui-Dong Yao, Yi-Min Shu, Sen-Lin Shi, Zhao-Feng Peng, Wen-Yan Song, Hai-Xia Jin, Ying-Pu Sun

**Affiliations:** 1 Center for Reproductive Medicine, The First Affiliated Hospital of Zhengzhou University, Zhengzhou, People's Republic of China; 2 Program of Reproductive and Stem Cell Biology, Department of Obstetrics and Gynecology, Stanford University School of Medicine, Stanford, California, United States of America; Institute of Zoology, Chinese Academy of Sciences, China

## Abstract

As one of the non-classical major histocompatibility complex(MHC)-1 antigens, Human Leukocyte Antigen G (*HLA-G*), has been suggested as a prognostic marker to identify the embryo developmental potential. In the present study, we investigated the potential roles of *HLA-G* in human spermatogenesis and early embryonic development. Quantitative real-time PCR analysis revealed that *HLA-G*'s expression was increased with increased Johnsen score in testicular tissues. There was no significant difference in *HLA-G* mRNA expression between testicular tissues with Johnsen score of 8–9 and normal sperm from ejaculated semen. *HLA-G* mRNA expression was detected in human zygotes, embryos and blastocysts but not in unfertilized oocytes. In testicular tissues where sperm was obtained by testicular sperm extraction (Johnsen score was 8 to 9), there were no correlations between *HLA-G* mRNA expression and fertilization, cleavage and high-quality embryo rates. At 48–72 h post-fertilization, *HLA-G* expression was higher in fast growing embryos. *HLA-G* specific siRNA injection into zygotes not only slowed down embryonic cleavage rate at 48 h post-fertilization, but also down-regulated the expression of embryo metabolism related gene (*SLC2A1*) and cell cycle-regulated gene (*CCND2*). Taken together, our findings suggested that *HLA-G* plays significant roles in human spermatogenesis and early embryonic development.

## Introduction

Human leukocyte antigen G (*HLA-G*) is a non-classical major histocompatibility complex (MHC) class I antigen characterized by specific tissue distribution and gene variation. *HLA-G* proteins can be expressed as seven distinct isoforms, by means of an alternative splicing from a single primary transcript. Four isoforms are membrane-bound proteins (*HLA-G1, -G2, -G3* and *-G4*), and the other three isoforms are soluble proteins (*HLA-G5, -G6 and -G7*). Membrane-bound *HLA-G* can be modified into soluble isoforms [1], [2]. As an important immunomodulatory molecule, *HLA-G* inhibits cytotoxic activity of T cell, natural killer (NK) cell lysis and cell proliferation. *HLA-G* regulates the maturation, migration, transportation of dendritic cells and cross talk between T cell and NK cell. *HLA-G* also plays a fundamental role in maternal-fetal tolerance by balancing cytokines Th1/Th2 [3]. The functions of *HLA-G* are fulfilled by inhibitory receptors expressed on the cell surface, such as ILT-2 (immunoglobulin-like transcript-2/LILRB1), ILT-4 (Ig-like transcript-4/LILRB2) and KIR2DL4 (killer inhibitory receptor) [4], [5], [6]. Except for its immune function, the non-immune function of *HLA-G* in reproduction has been discovered in recent years [2], [7], [8], [9].

So far only a few studies have focused on *HLA-G*'s expression in male reproductive system, in which *HLA-G* expressed in human sperm [10], prostate [12], testicular tissues and seminal plasma [13], and also testicular tissues and sperm of rhesus monkey [11]. It is still controversial regarding the expression of *HLA-G* in precursor germ cells and spermatids. *HLA-G* has been detected in immune-privileged sites such as the placenta and the cornea [14]. Furthermore, previous studies have shown that seminal plasma had an immune logical ‘priming’ effect in the female before embryo implantation [13], [15]. Therefore, it is hypothesized that *HLA-G* not only expresses in male reproductive tissues, but also plays an important role in the regulation of male fertility.

The expression of *HLA-G* was first detected in mature sperm by Chiang et al. with RT-PCR technique [10]. Ryan et al. investigated the expression of Mamu-AG, a homolog gene to human *HLA-G* in rhesus monkey [11]. According to their observation, the expression of Mamu-AG mRNA in seminiferous tubules was strikingly cell specific and its expression was relatively weaker in germ cells and sertoli cells compared with that in spermatocytes. However, it was difficult to detect Mamu-AG mRNA expression in interstitial cells around testis. Langat et al. detected *HLA-G1, -G2, -G5* and *-G6* specific mRNAs in normal prostate glands and prostate adenocarcinomas. *HLA-G5* protein was detectable in the cytoplasm of tubuloglandular epithelia and in glandular secretions, but not in prostate adenocarcinomas by immunohistochemistry [12]. Through ELISA technique, soluble *HLA-G* (*sHLA-G*) was found in human seminal plasma samples by Larsen et al.; however, there is a high variability in seminal *sHLA-G* concentrations between male donors. In addition, positive signals of HLA-G were detected in normal testis and in epididymal tissue by immunohistochemistry staining [13].

Similar to the *HLA-G* expression in testicular tissues, inconsistent information existed regarding the HLA expression in oocytes and preimplantation embryos. Earlier research claimed that HLA type I and II were not expressed in human oocytes and preimplantation embryos [16], [17]. However, Roberts et al. [18] and Jurisicova et al. demonstrated the *HLA-G* mRNA expression in the whole developmental stages from human oocytes to blastocysts by RT-PCR [8],[9]. Results from more recent studies showed the percentage of *HLA-G* mRNA expression increased with the developmental stage of embryos [8], [9], [19]. In a recent study containing a total of 4212 embryos, the proportion of *sHLA-G* positive embryos increased from 11% in zygotes to 21% in 2–9-cell embryos and 30% in embryos with greater than 10 cells respectively [2]. In addition, the authors observed the overall pregnancy rate and the pregnancy rate after single embryo transfer were significantly improved when *sHLA-G* positive embryos were transferred. However, the underlying mechanisms remain largely unknown.

Our results from this study demonstrated significant roles of *HLA-G* on both human spermatogenesis and embryonic development. We compared the *HLA-G* mRNA expression in testicular tissues with different spermatogenic ability, unfertilized oocytes and different stages of human embryos. Association of *HLA-G* expression in testicular tissues with Johnsen score of 8–9 with oocyte fertilization, embryo cleavage and high quality embryo rates were also investigated. The underlying mechanisms of *HLA-G* on human early embryonic development were also demonstrated by injecting *HLA-G* specific siRNA into zygotes.

## Materials and Methods

### Patient population

This study was approved by the Ethics Committee of the First Affiliated Hospital of Zhengzhou University. Donated human gametes, embryos and testicular tissues were used with written informed consent received from patients seeking infertility treatment at the Center for Reproductive Medicine in the First Affiliated Hospital of Zhengzhou University from September 2011 to June 2013.

### Collection of testicular tissues, RNA extraction and reverse transcription (RT)

Testicular sperm extraction (TESE) was performed prior to treatment with assisted reproduction, with part of the testicular tissues being sent to pathologic department for pathological analysis and the rest being frozen for future fertility treatment. Spermatogenesis was evaluated with the Johnsen scoring system [20], where all tubular sections in each section of the testicular biopsy were evaluated systematically and each was given a score from 1 to 10. The criteria were as follows: 10, complete spermatogenesis and perfect tubules; 9, many spermatozoa present but disorganized spermatogenesis; 8, only a few spermatozoa present; 7, no spermatozoa but many spermatids present; 6, only a few spermatids present; 5, no spermatozoa or spermatids present but many spermatocytes present; 4, only a few spermatocytes present; 3, only spermatogonia present; 2, no germ cells present; 1, neither germ cells nor Sertoli cells present. A total of 46 testicular tissues were collected in the study, among which 7 were from patients with non-obstructive azoospermia (Johnsen score 2–7), 37 were from patients with obstructive azoospermia or hypospermatogenesis (Johnsen score 8–9), and 2 were from patients with difficulty in semen collection on the day of oocyte retrieval (Johnsen score 10).

Frozen testicular tissues were diced and fully grinded in liquid nitrogen, and then mixed with lysis buffer of PureLink RNA Mini Kit (Life Technologies, Inc., Gaithersburg, MD). RNA was extracted using the PureLink RNA Mini Kit. The extraction was treated with DNase I (50U) at 37°C for 30 min to remove the possible contamination with DNA. Finally, the RNA sample was dissolved in 15 μl RNase-free water. The concentration of extracted total RNA was measured by spectrophotometry. Total RNA extraction was reverse-transcribed by High Capacity cDNA Reverse Transcription Kits (Life Technologies) as described in the product manual.

### Sperm preparation and RNA extraction

A total of 9 normal semen samples were included in this study. These couples received assisted reproductive therapy exclusively due to female factors. After 30 min liquefaction at room temperature, semen analysis was performed for the assessment of total sperm count, sperm motility and morphology. The mean age of the male patients are 33.5±5.9 years. To purify the semen sample, seminal plasma was separated from the sperm by centrifuging at 350 g for 15 min in 45% and 90% of sperm Grad (Vitrolife Sweden AB, Goteborg, Sweden) solution made by G-IVF (Vitrolife) supplemented with 10% human serum albumin (HSA) solution (Vitrolife).

The sperm pellet was then washed twice by suspension in G-IVF solution, after which the supernatant was discarded and the sperm pellet was subsequently suspended in fresh G-IVF solution. Left the centrifuge tube inclined at 30° for 15 min for sperm swim-up. The supernatant containing progressively motile spermatozoa was used for IVF, with the residual being centrifuged to remove the supernatant for RNA extraction. Sperm RNA was extracted with PureLink RNA Mini Kit, measured and reverse-transcribed as described above.

### Collection of oocytes, 1-cell zygotes, embryos and blastocysts, RNA extraction and RT

Human oocytes and embryos were cultured in a humidified 37°C incubator with 6% CO_2_, 5% O_2_, and 89% N_2_. Immature oocytes were collected from ICSI cycles, where metaphase I (MI) and germinal vesicle (GV) stage oocytes were *in vitro* cultured to the metaphase II stage. The *in vitro* matured oocytes were cryopreserved and stored in liquid nitrogen until use. Due to the difficulty in obtaining normally fertilized embryos for research, clinical useless embryos, including three-pronuclear (3PN) zygotes the day after oocyte insemination and poor-quality embryos at 72 h post-fertilization were collected and used in this study. To obtain embryos at different developmental stages, some 3PN zygotes were cryopreserved 16–18 h post-fertilization (day 1) and the rest were cultured until 44–46 h (day 2) or 67–69 h (day 3) post-fertilization prior to cryopreservation. Poor-quality embryos unsuitable for either transfer or cryopreservation at 72 h post fertilization were cultured to the blastocyst stage using G-2 (Vitrolife) supplemented with 6% HSA solution (Vitrolife). Blastocysts were frozen 116–118 h post-fertilization and stored until use.

After thawing, oocytes, 1-cell zygotes, cleavage stage embryos and blastocysts used for RNA extraction were first cultured for 2–3 h before being transferred to 4% pronase (Sigma, St. Louis, MO, USA) solution made by G-MOPS (Vitrolife) supplemented with 5% HSA. Zona-free oocytes, zygotes, embryos and blastocysts were washed several times with G-MOPS to remove the remaining zona pellucida, granulosa cells and spermatozoa. We used Embryo Vitri System-Freezing and Warming medium Kits (Origio Biotech, Denmark) for vitrification and warming. RNA was extracted with RNeasy Micro Kit (Qiagen, Valencia, CA, USA) according to the manufacturer's instructions, and quantified by spectrophotometry. Superscript III 1^st^ strand kits (Life Technologies) were used for RT according to the product manual.

### Microinjection and embryo culture

After warming, the vitrified 3PN zygotes were cultured in G-1 medium for 2–3 h before intracytoplasmic siRNA microinjection as described by Homer et al. [21]. All operations were performed in G-MOPS medium covered with paraffin oil and maintained at 37°C. SiRNA of *HLA-G* and scrambled siRNA (Santa Cruz Biotechnology, Inc., Santa Cruz, CA) are si-*HLA-G* and si-CONTROL respectively. Concentrations of siRNAs are 10 μM and the injected solution was equivalent to ∼5% of the total zygote volume. After microinjection, the zygotes were transferred to G-1 medium for culture a humidified 37°C incubator with 6% CO_2_, 5% O_2_, and 89% N_2_. Embryonic development was evaluated 48 h and 72 h post-fertilization respectively.

### Quantification of *HLA-G*, *PLAC8*, *CCND2*, *SLC2A1* and *GAPDH* mRNA transcripts by real-time PCR

Real-time PCR was performed as described previously [22]. After cDNA synthesis, real-time PCR was run in an Applied Biosystems 7500 fast real time PCR system (Applied Biosystems, Foster City, CA,USA) using a SYBR select master mix kit (Applied Biosystems) and the reaction system is 20 μl. All of the primers were designed with Primer-BLAST in NCBI (http://www.ncbi.nlm.nih.gov/tools/primer-blast/). The primers spanned two different exons to avoid the possible DNA resources, and the target-specificity was confirmed by BLAST searching. Primers used for the amplification of human *CCND2*, *HLA-G*, *PLAC8* and *SLC2A1* are listed in Table S1. PCR was carried out with the following conditions: 50°C for 2 min, 95°C for 2 min. The cDNA was amplified for 40 cycles, with each cycle consisting of 95°C for 15 s, 58°C for 15 s and 72°C at 1 min. The amplification products were separated on 3% agarose gels to determine if non-specific products were produced. The melting curves for a number of PCR products are single-peaked. Data was analyzed by using the comparative ΔΔCt method [23], and the mRNA expressions of *CCND2*, *HLA-G*, *PLAC8* and *SLC2A1* were normalized to the *GAPDH* expression. The experiments were repeated three times.

### Statistical analysis

Results were expressed as means ±SEM with 3 repeated experiments and Student's *t* test was used for 2 groups of statistical analysis. The expression of *CCND2*, *HLA-G*, *PLAC8* and *SLC2A1* mRNA was normalized to that of *GAPDH* mRNA expression. Fertilization rate was calculated by dividing the number of zygotes by total number of oocytes receiving ICSI. Cleavage rate means the number of cleaved embryos 48 h post-fertilization divided by the total number of cultured zygotes. High-quality embryo rate was defined as the percentage of high-quality embryos 72 h post-fertilization. The ratios were analyzed by the Chi-square test. A *P*-value of less than 0.05 was considered statistically significant.

## Results

### *HLA-G* expressions are different in testicular tissues with different spermatogenic abilities

**Figure 1:**
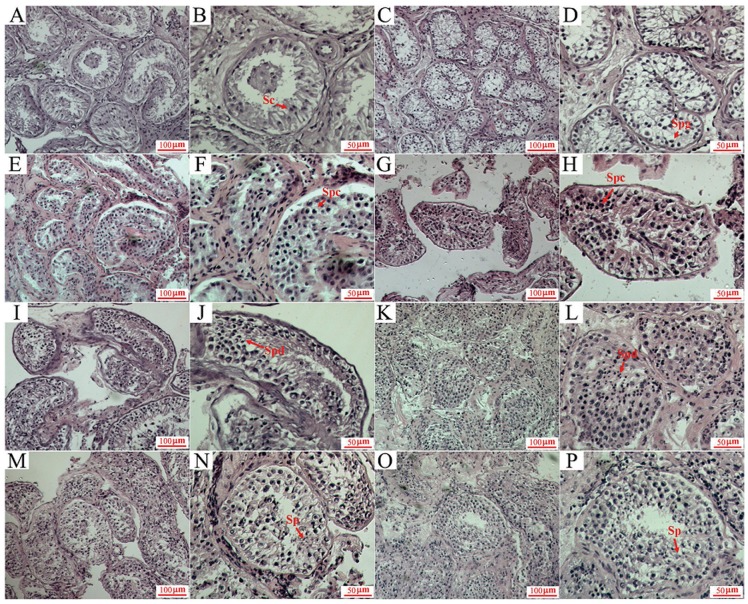
HE staining of testicular tissues with different spermatogenic conditions. (A–B) Only sertoli cells were observed in seminiferous tubules. Johnsen score was 2.(C–D) Sertoli cells and few spermatogonia were seen in seminiferous tubules. Johnsen score was 3.(E–F) Sertoli cells, spermatogonia and few spermatocytes but not spermatids or mature spermatozoa were found in seminiferous tubules. Johnsen score was 4.(G–H) Spermatogenic cells at different stages were observed in seminiferous tubules, but arranged irregularly. Spermatids and mature spermatozoa are not seen. Johnsen score was 5.(I–J) Spermatogenic cells were disorganized in seminiferous tubules, but the number decreased significantly; few early spermatids were seen in seminiferous tubules, without any spermatozoa. Johnsen score was 6. (K–L) Spermatogenic cells and many early spermatids were found, but with no late spermatids and spermatozoa. Johnsen score was 7.(M–N) Few of late spermatids and mature spermatozoa were found in seminiferous tubules. Johnsen score was 8.(O–P) Many late spermatids and mature spermatozoa can be seen. Johnsen score was 9. Figures of right side were amplified from the left ones. Sc, Sertoli cell. Spg, Spermatogonia. Spc, Spermatocyte. Spd, Spermatid. Sp, Sperm. Scale bar: 50 μm or 100 μm as indicated in the right-down corner.

**Figure 2:**
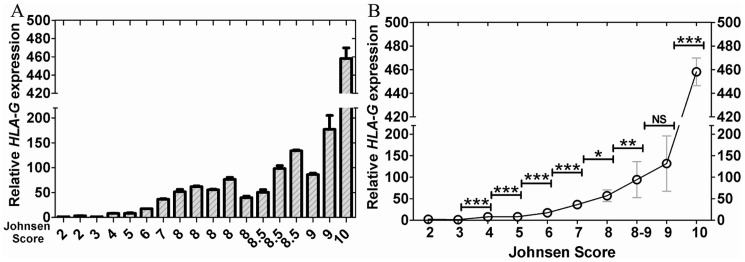
Differential expressions of *HLA*-G in testicular tissues with various spermatogenic conditions. Expressions of *HLA-G* mRNA in testicular tissues with various Johnsen scores (A–B). *HLA-G* expressions were normalized with the reference gene *GAPDH* and with the sample which Johnsen score is 2. NS means no significant. *, ** and *** represent *P*<0.05, *P*<0.01 and *P*<0.001 respectively.

Hematoxylin and eosin (HE) staining of testicular tissues was shown in Fig.1. A total of 17 testicular tissues were selected from the male patients aged 29.1±5.8 years. Except for testicular tissues from azoospermia patients, two testicular tissues retrieved from normospermic patients due to difficulty in semen collection on the day of oocyte retrieval were also examined for HLA-G mRNA expression (Fig. 2A and B). Both patients showed normal sperm count and sperm motility and Johnsen score was indicated as 10. Levels of HLA-G gene expression were quite different in testicular tissues with various Johnsen scores (Fig. 2A). As shown in Fig. 2B, there was a trend to increased expression of *HLA-G* mRNA with an increased Johnsen score, with the highest level of *HLA-G* mRNA expression being seen in testicular tissues with a Johnsen score of 10.

### *HLA-G* mRNA expression in sperm ejaculated from patients with normal spermatogenic abilities

**Figure 3:**
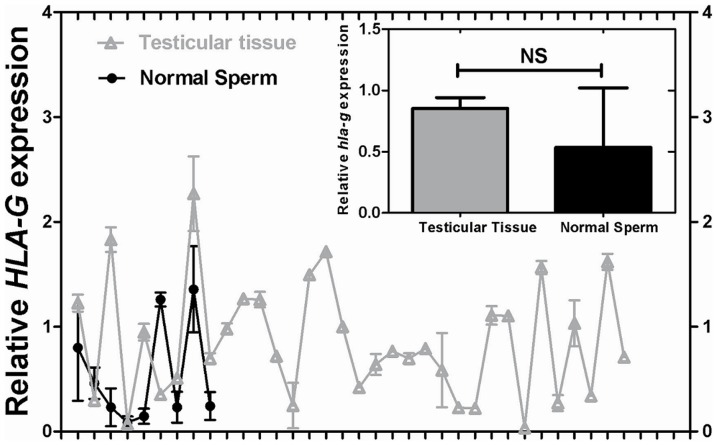
*HLA-G* mRNA expression between sperm from normal semen and testicular tissues retrieved through testicular sperm extraction. A total of 9 normal semen samples and 34 testicular tissues with Johnsen scores of 8–9 were enrolled. Expressions of *HLA-G* mRNA in normal sperm were normalized with *GAPDH*, and *HLA-G* expressions were standardized with one of the testicular tissue in which *HLA-G* expression was at the middle level. The small picture shown at top right corner demonstrated no significant difference of *HLA-G* mRNA expression between normal sperm and testicular tissues. NS represents no significant difference.

We further compared the *HLA-G* mRNA expression in testicular tissues from azoospermia patients with Johnsen score of 8–9 and those with normal semen parameters. Nine tubal factor infertility couples without male chromosomal or basic endocrine disorders were enrolled. The average age of the male patients was 33.5±5.9 years. Semen analysis showed that sperm concentration was 50–100×10^6^/ml, and the percentage of the sperm with forward movement was more than 45%. In addition, the oocyte fertilization rate was 91.8±19.7% after *in vitro* fertilization. We did not to observe statistic difference in *HLA-G* mRNA expression between sperm from normal semen and testicular tissues with Johnsen score of 8–9 (*P*>0.05) (Fig.3). In addition, there was no association between *HLA-G* mRNA expression and oocyte fertilization, embryo cleavage or high-quality embryo rate (data not shown).

### *HLA-G* expression in testicular tissues had no impact on oocyte fertilization, embryo cleavage or high-quality embryo rates after ICSI treatment

**Figure 4:**
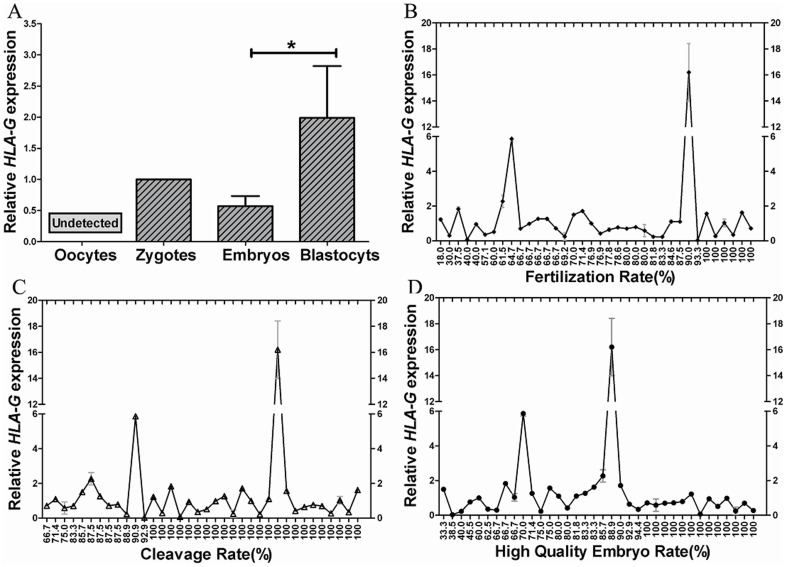
Relationship between *HLA-G* expression in testicular tissues and oocyte fertilization, embryo cleavage or high-quality embryo rate after ICSI treatment. Except for oocytes, the expression of *HLA-G* mRNA were detected in 3PN zygotes, embryos and blastocysts were detected (A). *HLA-G* mRNA expression in tissues from testicular extraction had no effect on fertilization rate (B), embryo cleavage rate (C), or high-quality embryo rate (D) after ICSI treatment. *HLA-G* mRNA expression was normalized with *GAPDH*, and all of the samples were standardized with the data used in Fig. 3. *HLA-G* mRNA expression was normalized with *GAPDH*, and *HLA-G* expression in embryos and blastocysts was standardized with that in zygotes. * means *P*<0.05.

*HLA-G* mRNA expression was much higher in testicular tissues with higher ability of spermatogenesis, and the highest expression was in seminiferous tubules that tend to produce mature sperm. Based on the above results, we hypothesized that *HLA-G* might have significant roles on embryonic development after oocyte fertilization. Total RNAs were extracted from oocytes, zygotes, embryos and blastocysts, and the *HLA-G* mRNA expressions were analyzed. Our results showed that *HLA-G* mRNA expression was detected in zygotes, embryos and blastocysts but not in oocytes (Fig. 4A). After normalization, there was a trend to lower *HLA-G* expression in cleavage stage embryos; however, this difference was not statistically significant. We also observed that *HLA-G* expression in blastocysts was significant higher than that in cleavage stage embryos (*P*<0.05).

Since the *HLA-G* expression were not detected in unfertilized oocytes, we hypothesized that *HLA-G* expression might be correlated with oocyte fertilization, embryo cleavage and high-quality embryo rate. Thirty-six couples undergoing ICSI with TESE sperm were enrolled. The average ages of female and male patients were 28.1±4.5 and 29.9±5.6 years, respectively. Endometriosis, polycystic ovarian syndrome (PCOS), premature ovarian failure (POF), and other infertility factors were excluded from female patients. The average number of oocytes retrieved was 11.7±5.5, with 10.4±4.8 mature oocytes being used for ICSI. Testicular tissues with Johnsen score of 8–9 or with mature sperm on the day of oocyte retrieval were used. The results showed that *HLA-G* expression was not correlated with oocyte fertilization, embryo cleavage, or high-quality embryo rate (Fig. 4B, C and D).

### *HLA-G* gene silencing impaired the embryonic development

As shown above, *HLA-G* mRNA expression was increased with an increased Johnsen score and there was no significant difference in *HLA-G* expression between mature semen sperm and testicular tissues with sperm. *HLA-G* expression was also detected in zygotes and embryos but not in unfertilized oocytes. Our results demonstrated that the high quantity of *HLA-G* mRNA at the late stages of spermatogenesis may closely correlate with spermatogenesis and embryonic development post-fertilization.

**Figure 5:**
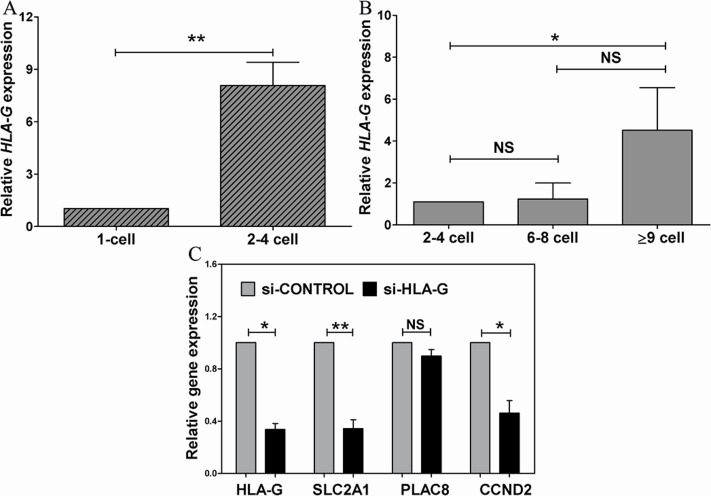
Gene expression in *in vitro* cultured and *HLA-G* silenced embryos. (A) Expressions of *HLA-G* mRNA in one-cell zygotes and 2-to 4-cell embryos at 48 h post-fertilization. (B) *HLA-G* mRNA expression in embryos with different numbers of blastomeres at 72 h post-fertilization. *HLA-G* mRNA expression was normalized with *GAPDH*, and standardized with expression in one-cell zygotes 48 h post-fertilization (A) and 2–4 cell embryos 72 h post-fertilization (B). (C) The expressions of *HLA-G*, *SLC2A1*, *PLAC8* and *CCND2* were normalized with *GAPDH*, standardized with the expressions in si-CONTROL group. NS means no significant difference. * and ** represent *P*<0.05 and *P*<0.01 respectively.

The present study confirmed *HLA-G* mRNA expression in early human embryos. Based on our observation, there was a differential expression of HLA-G mRNA between 2–4-cell stage embryos and those arrested zygotes. Investigation on one-cell zygotes and 2–4 cell stage embryos demonstrated that *HLA-G* mRNA expression in normally developing cleavage stage embryos was significantly higher than in one-cell zygotes (*P*<0.01) (Fig. 5A). At 72 h post-fertilization, no significant difference of *HLA-G* mRNA expression was detected between slowly developing or arrested embryos with 2–4 cells and normally developed embryos with 6–8 cells. However, the *HLA-G* mRNA expression in embryos with 2–4 cells was significantly lower than embryos with more than or equal to 9 cells (*P*<0.05) (Fig. 5B). These results further demonstrated that *HLA-G* was really involved in embryonic development.

Microinjection of *HLA-G* specific siRNA into 3PN zygotes at 20 h post-fertilization is a useful method to investigate the effects of *HLA-G* on embryonic development. The silencing effect was confirmed by significantly decreased *HLA-G* mRNA expression in day 2 embryos at 24 h after siRNA injection compared with control embryos injected with scrambled siRNA (*P*<0.05) (Fig. 5C). No statistic difference in the percentage of cleaved embryos was detected 48 h post-fertilization between *si-HLA-G* group and the si-Control group (*P*>0.05) (Table 1). Moreover, the percentages of cleaved embryos in both groups were more than 90%, indicating that the injection procedure per se did not harm subsequent embryonic development (Table 1). However, observation on day 3 embryos at 72 h post-fertilization showed that the percentage of slowly cleaved embryos in control group was significantly lower than that in *HLA-G* silencing group (40.4% vs 58.7%, *P*<0.05), and the percentage of normally cleaved embryos in *HLA-G* silencing group was significantly lower than that in control group (41.0% vs 59.6%, *P*<0.05) (Table 1), demonstrating that the down regulation of *HLA-G* mRNA by HLA-G gene silencing decreased the embryo cleavage speed.

**Table 1:** Embryo cleavage after microinjection of *HLA-G* siRNA.

		si-CONTROL	si-*HLA-G*	P
**48 h embryos**	**No Cleavage (%)**	**9.62(5/52)**	**6.52(3/46)**	**NS**
	**Cleavage (%)**	**90.38(47/52)**	**93.48(43/46)**	**NS**
**72 h embryos**	**Slow Cleavage (%)**	**40.38(21/52)**	**58.70(27/46)**	**<0.05**
	**Normal Cleavage (%)**	**59.62(31/52)**	**41.30(19/46)**	**<0.05**

NS means no significant difference.

“No Cleavage” means one-cell zygote without cleavage 48 h post-fertilization; “Cleavage” means embryos with 2–4 blastomeres 48 h post-fertilization; “Slow Cleavage” means embryos with less than or equal to 5 blastomeres 72 h post-fertilization; “Normal Cleavage” means embryos with more than or equal to 6 blastomeres 72 h post-fertilization.

Several embryonic developmental related genes were also studied. Our results showed that the metabolism-related gene *SLC2A1* and cell cycle regulating-related gene *CCND2* were significantly down regulated after *HLA-G* silencing. However, although *PLAC8* has many functions similar to *HLA-G* in reproduction, there was no significant difference in *PLAC8* expression in embryos after *HLA-G* silencing (Fig. 5C).

## Discussion

Testicular tissue scoring system (Johnsen's scoring) has been used to evaluate the quality and quantity of seminiferous epithelium established since 1970 [20]. We investigated *HLA-G* expression in testicular tissues with Johnsen scores of 2–9 (Fig. 1). Our results demonstrated that *HLA-G* mRNA levels in testicular tissues with spermatocytes were significantly higher than those with only sertoli cells and/or spermatogonia. Considering that the spermatocytes represent the first differentiation step of spermatogonia into spermatids and ultimately mature spermatozoa, *HLA-G* might be involved in this differentiation process, or play vital roles in supporting the following spermatogenesis process.

To our knowledge this is the first study reporting that the expression of *HLA-G* mRNA is increased with the value of Johnsen score in human testis, indicating the critical role of *HLA-G* in spermatogenesis. To further investigate the regulation of *HLA-G* in spermatogenesis, different types of cells, including sertoli cells, spermatogonia, spermatocytes and spermatids should be isolated to find out the expression patterns of *HLA-G* mRNA and protein. Considering the molecular characteristics of *HLA-G*, investigating the expressions of non-coding RNAs(such as miR-152, miR-148a and miR-148b) in different types of testicular tissues are also very important, for that these miRNAs are involved in the post-transcriptional regulation of *HLA-G* expression[24].

Expressions of *HLA-G* mRNA were also detected in human sperm purified from ejaculated semen, and the levels of *HLA-G* mRNA expressions in sperm were matching with that in testicular tissues containing mature sperm (Johnsen score 8–9), indicating that during the late stages of spermatogenesis, parts of the abundantly synthesized *HLA-G* mRNA may entering into the oocyte during the fertilization process. We failed to observe obvious correlations between the expressions of *HLA-G* mRNA in testis tissue with mature sperm and fertilization, cleavage and high quality embryo rate, which might be due to the many factors affecting the fertilization, embryo cleavage and formation of high quality embryo, such as stimulation protocol, oocyte quality, culture methods, and so on. Therefore, further studies should include more cycles and perform more detailed categorizations to reveal the relationship between the expressions of *HLA-G* mRNA and fertilization rate, embryo cleavage, etc.

Our results showed that the expression of *HLA-G* mRNA can be detected in zygotes and embryos or blastocysts, but not in unfertilized oocytes. However, Jurisicova et al reported that the expression of *HLA-G* mRNA were detectable in unfertilized human oocytes [9]. By analyzing between-study differences, we realized that phenol-chloroform extraction of total RNAs and RT-PCR amplification methods were used in their study. In our study, total RNAs were extracted by using widely used commercially available RNA extraction kits, and the obtained total RNAs were further treated with DNase I to eliminate the possible genomic contamination, which might be one of the reasons for the discrepancy. Another possible reason is the low abundance of *HLA-G* mRNA in unfertilized oocytes. Because *HLA-G* is associated with implantation and protection of the allogenic fetus [25], [26], expression of *HLA-G* mRNA in blastocysts is significantly higher than that in cleavage stage embryos.

Except for the widely used morphological evaluating system, researchers are now trying to use non-invasive methods to predict the embryo developmental potential, such as determining the compositions and concentrations in embryo culture media [2], [8]. Embryonic secretion of *sHLA-G* has been postulated to be one of the most promising markers of embryonic development potential [2], [8], [9]. Jurisicova et al. (1996) were the first to reveal the existence of an important correlation between *HLA-G/sHLA-G* and embryo implantation in assisted reproduction [8], [9]. A recent multi-center research showed that the proportion of *sHLA-G* positive embryos increased from 11% in zygotes to 21% in 2–9-cell embryos and 30% in embryos with more than 10 cells [2].

Injection of HLA-G gene specific siRNA into the cytoplasm of 3PN zygotes at 24 h post-injection significantly down-regulated the expressions of HLA-G mRNA, indicating that a similar RNA inference mechanism operating in somatic cells also existed in human zygotes or cleavage stage embryos. As shown in our results, although embryo cleavage at 48 h post-fertilization was not significantly affected by injecting with *HLA-G* specific siRNA, the levels of *HLA-G* mRNA expressions were significantly higher in cleaved embryos than those arrested ones. One of the possible explanations is that a certain time period is needed for down-stream effects after siRNA injection. At 72 h post-fertilization, not only did embryos with *HLA-G* silencing develop slower than those in control (*P*<0.05), but also the percentage of normally developing embryos was significantly lower in embryos injected with *HLA-G* specific siRNAs than those injected with scrambled siRNAs (*P*<0.05). Our results were consistent with a recently published report by Sun et al. [7], where the percentage of arrested 3PN embryos was significantly increased in embryos with *HLA-G* silencing by using a virus-based transfection method. The authors also observed decreased number of blastomeres in embryos with *HLA-G* silencing [7]. Results from theirs and our own study, therefore, demonstrated that *HLA-G* may be an important molecule in regulating early embryonic development.

In order to explore the possible mechanisms of *HLA-G* in regulating human embryonic development, several embryo developmental related genes were analyzed [27], [28], [29]. *SLC2A1* is one of the primary glucose transporters in the preimplantation embryos [29], [30]. Prior to embryo compaction, *SLC2A1* is localized primarily to the nucleoli and nuclear membranes. The levels of *SLC2A1* mRNA and protein are significantly increased post-compaction. *SLC2A1* is primarily localized to the basolateral membrane of trophectoderm cells and the plasma membrane of inner cell mass cells at this time, permitting the shuttling of glucose from the cavity to the inner cell mass [31]. The localization of *SLC2A1* coincides with the energy preference in the developing embryos from the utilization of pyruvate pre-compaction to the utilization of glucose post-compaction. In this study, we also observed lower levels of *SLC2A1* mRNA and lower rates of embryo development in *HLA-G* silenced embryos. *CCND2* (cyclin D2), an important cell cycle regulator, functions as a cell cycle clock apparatus during G1 phase of the mammalian cell cycle. Entering into the S phase from G1 phase is positively controlled by *CCND2* [28]. *CCND2* is a rate-limiting activator of cell cycle progression [28], [32] that plays pivotal roles in ovarian granulosa cell proliferation. The expression of *CCND2* mRNA was significantly decreased in embryos with *HLA-G* silencing. *PLAC8*, an invasion specific gene, was found to be up-regulated in embryo biopsies that ended up with calf delivery [33], and expressed significantly higher in endometrium of pregnant than non-pregnant [29]. Considering that many functions of *PLAC8* are very similar to *HLA-G* in reproduction, the levels of *PLAC8* mRNA were also measured. Though the levels of *PLAC8* mRNA are decreased in embryos with *HLA-G* silencing, no significant differences were observed between treatment groups and control groups. Therefore, through analyzing the embryo developmental-related genes expression, we presume that the regulation of embryo metabolism and cell cycle progression are affected by *HLA-G* silencing.

In conclusion, the present study provides the first evidence of the differential expression of *HLA-G* mRNA in testicular tissues with different spermatogenic ability (according to Johnsen scoring system) and different stages of human embryos. Silencing of *HLA-G* not only decreased embryonic development, but also mRNA levels of embryo metabolic related gene, *SLC2A1* and cell cycle regulated gene, *CCND2*. Taken together, our findings suggested that *HLA-G* plays significant roles in human spermatogenesis and early embryonic development.

## Supporting Information

Table S1 Specific primers used for real-time PCR.
(DOC)
