# Supplementary material for: Expression and Potential Roles of HLA-G in Human Spermatogenesis and Early Embryonic Development
Source: PLoS One. 2014 Mar 25;9(3):e92889. doi: 10.1371/journal.pone.0092889 (PMC3965489; doi:10.1371/journal.pone.0092889)
Supplement: Table S1 — Specific primers used for real-time PCR. (DOC) [file pone.0092889.s001.doc]

**Table S1. Specific primers used for real-time PCR**

| Gene | Primer | Sequences | Amplicon size(bp) |
| --- | --- | --- | --- |
| *CCND2* | Forward | 5’-TACCTGGACCGTTTCTTGGC-3’ | 153 |
|  | Reverse | 5’-AGGCTTGATGGAGTTGTCGG-3’ |  |
| *HLA-G* | Forward | 5’-CTGGTTGTCCTTGCAGCTGTAG-3’ | 80 |
|  | Reverse | 5’-CCTTTTCAATCTGAGCTCTTCTTTCT-3’ |  |
| *PLAC8* | Forward | 5’-TGTCTGTGTGGAACAAGCGT-3’ | 196 |
|  | Reverse | 5’-AGGCATGTTTGCATTGACTCAC-3’ |  |
| *SLC2A1* | Forward | 5’-TCACTGTCGTGTCGCTGTTT-3’ | 214 |
|  | Reverse | ACGATGAACCATGGGATGGG-3’ |  |
| *GAPDH* | Forward | 5’-CTGCACCACCAACTGCTT-3’ | 105 |
|  | Reverse | 5’-TTCTGGGTGGCAGTGATG-3’ |  |
